# BRD4 contributes to LPS-induced macrophage senescence and promotes progression of atherosclerosis-associated lipid uptake

**DOI:** 10.18632/aging.103200

**Published:** 2020-05-11

**Authors:** Hui Wang, Haiping Fu, Ruigong Zhu, Xuan Wu, Xian Ji, Xuesong Li, Hong Jiang, Zhe Lin, Xin Tang, Shixiu Sun, Jiajing Chen, Xin Wang, Qingguo Li, Yong Ji, Hongshan Chen

**Affiliations:** 1 Key Laboratory of Cardiovascular and Cerebrovascular Medicine, School of Pharmacy, Nanjing Medical University, Nanjing, China; 2 Department of Cardiothoracic Surgery, The Second Affiliated Hospital of Nanjing Medical University, Nanjing, China; 3 Key Laboratory of Targeted Intervention of Cardiovascular Disease, Collaborative Innovation Center for Cardiovascular Disease Translational Medicine, Nanjing Medical University, Nanjing, China; 4 Faculty of Biology, Medicine and Health, The University of Manchester, Manchester, United Kingdom

**Keywords:** inflammation, senescence, BRD4, macrophage, gene expression

## Abstract

Aging is closely associated with atherosclerosis. Macrophages accumulate in atherosclerotic lesions contributing to the development and progression of atherosclerosis. Although atherosclerotic lesions are known to contain senescent cells, the mechanism underlying the formation of senescent macrophages during atherosclerosis is still unclear. In this study, macrophages with different origins were collected, including THP-1 macrophages, telomerase reverse transcriptase knock out (Tert^-/-^) mouse peritoneal macrophages, and human peripheral blood mononuclear cells (PBMCs). We found Lipopolysaccharide (LPS) could induce the formation of senescent macrophages, which was typified by the morphological changes, senescence-associated secretory phenotype (SASP) secretory, and persistent DNA damage response. Mechanistically, bromodomain-containing protein 4 (BRD4), a chromosomal binding protein related to gene expression, was found to play a key role in the pathological process, which could offer new therapeutic perspectives. Inhibition of BRD4 by siBRD4 or inhibitors such as JQ-1 or I-BET762 prevented the aging of macrophages and lipid accumulation in the LPS-induced senescent macrophages by decreasing expression of SASP in autocrine and paracrine senescence. These findings have significant implications for the understanding of the pathobiology of age-associated diseases and may guide future studies on targeted clinical drug therapy.

## INTRODUCTION

Atherosclerosis, a chronic cardiovascular disease stemming from the formation of plaque in the intima of arterial walls, is a leading cause of death worldwide [1, 2]. It is characterized by the accumulation of lipids in the intima, thickening of the artery wall, and narrowing of the vascular cavity, leading to a series of complications [3, 4]. Generally, atherosclerosis is induced by hypercholesterolemia, high blood pressure, and genetic risk factors. Meanwhile, it can be aggravated by obesity, diabetes, smoking, and a sedentary lifestyle [5–7].

The progressive development of atherosclerotic lesions is an age-dependent process [8]. Intrinsic vascular aging results in a range of pathophysiological consequences, including stroke, coronary heart disease, vascular rupture, and atherogenesis [9, 10]. Senescence is one of the main risk factors in most chronic severe diseases [11]. It has been reported that senescence is involved in cardiovascular disease and promotes the pathogenesis of atherosclerosis [12]. Chronic infections and aging diseases, such as atherosclerosis, are closely related. Lipopolysaccharide (LPS)-induced inflammation is closely associated with various aging disorders [13]. Systematic changes in the inflammatory response of aging organisms are highly likely to have an impact on aging diseases, such as atherosclerosis, Alzheimer's, and age-related macular degeneration [14, 15].

Activated macrophages are known to take part in atherosclerosis [16, 17]. The activated macrophages produce proteases and inflammatory cytokines [18], which aggravate the atherosclerotic process. However, the role of senescent macrophages in atherogenesis remains poorly understood. Although it has been reported in Science that senescent macrophages are associated with atherosclerosis, the underlying mechanism by which senescence of macrophages is induced in the pathologic process remains mostly elusive. Foamed macrophages with senescence markers coexist with inflammatory cytokines, chemokines, and metalloproteinases during atherosclerosis [19]. However, its underlying mechanism remains unknown. Inflammatory cell secretory molecules play an essential role in the progression of atherosclerosis and senescence [20–22]. As such, this study aims to elucidate the mechanism by which senescent macrophages stimulate inflammatory responses, thereby contributing to the development of novel drugs.

The bromodomain and extra-terminal domain (BET) proteins, comprised of BRD2, BRD3, BRD4, and BRDT, regulate the transcription of genes involved in many cell functions, including inflammation, apoptosis, and cell cycle progression [18, 23]. Considering that BET proteins are universally related to gene expression and were recently implicated in the pathogenesis of cardiovascular disease [24, 25], we investigated the role of BET proteins in infection-induced senescence of macrophage in the study. BRD4, one of the most characterized BET proteins in inflammation, binds to acetylated histones and non-histone proteins at enhancers and promoters to regulate gene expression programs [26, 27]. In this study, BRD4 was observed for the first time to be involved in this process. We furtherly demonstrate that BRD4 takes part in macrophage senescence and gives rise to SASP, resulting in the progression of atherosclerosis. Our findings suggest that siBRD4 may prevent the aging of macrophages, which is in turn beneficial for the inhibition of atherosclerosis.

## RESULTS

### LPS promotes senescence of macrophages with increased expression of BRD4 via NFκB pathway activation

**Figure 1:**
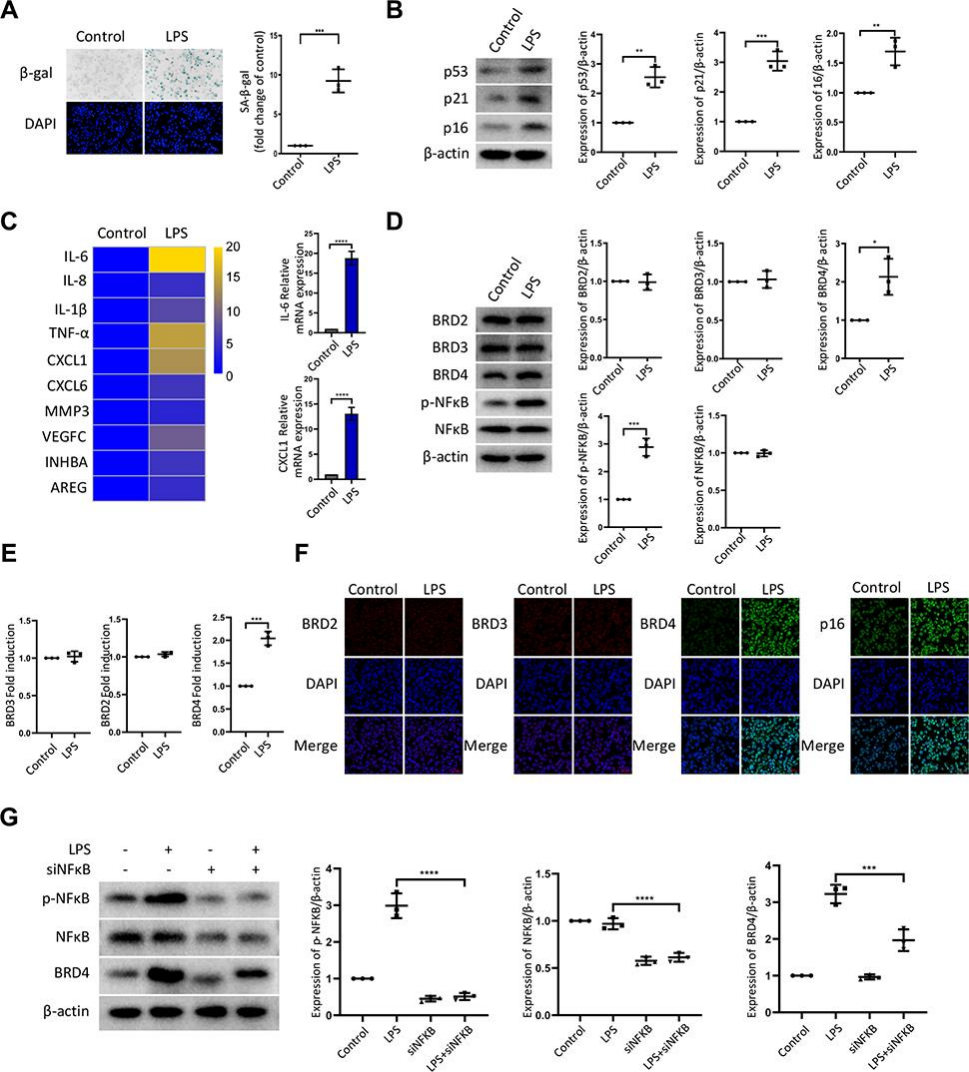
**LPS promotes senescence of macrophages with increased expression of BRD4 via NFκB pathway activation.** Cultures of THP-1 monocyte-derived macrophages were prepared and lipopolysaccharide (LPS, 1 μg·ml^−1^) was used to induce senescence. (**A**) Senescent THP-1 monocyte-derived macrophages induced by LPS were detected by senescence-associated β-galactosidase staining (SA-β-Gal staining). Scale bar, 50 μm. The quantification of the SA-β-gal positive cells is presented in the scatter plot. (**B**) Representative western blot and statistical data showing the protein levels of senescence markers p53, p21, and p16, with or without LPS. Actin was used as the loading control. (**C**) Real-time polymerase chain reaction (RT-PCR) was used to assess the expression of senescence-associated secretory phenotype (SASP) genes. (**D**) BRD2, BRD3, BRD4, pNF-kB and NF-kB levels were evaluated by western blotting. Actin was used for normalization. (**E**) mRNA levels of BRD2, BRD3, and BRD4 in THP-1 macrophages with or without LPS. (**F**) Immunofluorescence analysis of THP-1 macrophages with or without LPS stained for BRD2 (red), BRD3 (red), BRD4 (green), p16 (green), and Nuclei (DAPI, blue) were analyzed by confocal microscopy. (**G**) THP-1 macrophages were transfected with NF-κB-specific siRNA, followed by 1μg/ml LPS stimulation for 24 hours. Western blotting analysis and quantification of pNF-κB, NF-κB and BRD4 protein expression in THP-1 macrophages. Actin was used for normalization. The data all represent measurement data presented as the mean ± SD. The two groups were statistically analyzed using independent sample t-test. The experiment was repeated three times. Significant differences among the different groups are indicated as *p <0.05 vs. control; **p <0.01 vs. control; ***p <0.001 vs. control.

To establish the model of infection-induced macrophage senescence, we treated THP-1 macrophages with LPS for senescence-associated-β-galactosidase staining (SA-β-Gal staining). The number of cells positive for β-gal increased in response to LPS (Figure 1A). We found a corresponding upregulation in the protein expression of senescence markers p53, p21, and p16 (Figure 1B). At the mRNA level, we examined several genes associated with the senescence-associated secretory phenotype (SASP), including IL-6, IL-8, IL-1b, TNF-α, CXCL1, CXCL6, MMP3, VEGFC, INHBA, and AREG. The mRNA levels of these genes were elevated significantly by LPS treatment (Figure 1C). Nuclear factor-kappaB (NFκB) is a multifunctional transcription factor, which can be activated in the inflammatory response. Treatment with LPS resulted in a high expression of phosphorylated NFκB (p-NFκB) and BRD4, while the expression of BRD2 and BRD3 remained unchanged (Figure 1D, 1E). The expression of BRD2, BRD3, BRD4, and p16 in THP-1 cells by immunofluorescence was consistent with the findings above (Figure 1F). Strikingly, interference of the NF-κB pathway prevented the high expression of BRD4 induced by LPS stimulation (Figure 1G), demonstrating that LPS induced senescence of macrophages with high levels of BRD4 expression via the NFκB pathway activation.

### BRD4 is involved in macrophage senescence caused by inflammation

**Figure 2:**
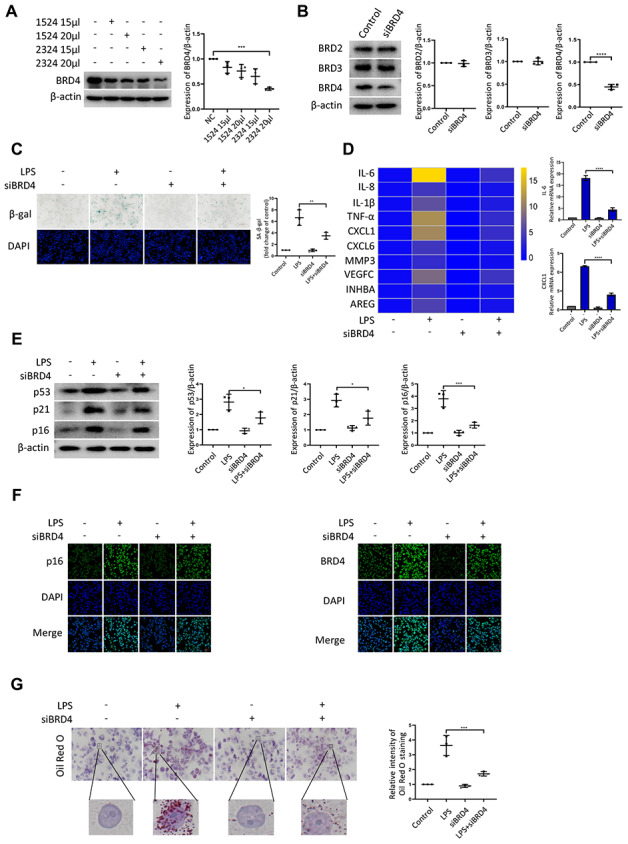
**BRD4 is involved in macrophage senescence caused by inflammation.** THP-1 macrophages were incubated with four different siRNAs for knockdown of BRD4. (**A**) BRD4 expression was evaluated by western blotting, as shown in the scatter plot. (**B**) Western blot analysis for BRD2, BRD3, and BRD4 protein expression. Actin was used for normalization. (**C**) SA-β-gal activity was analyzed after the knockdown of BRD4. The quantification of SA-β-gal positive cells is presented in the scatter plot. (**D**) Analysis of SASP genes mRNA levels in THP-1 macrophages. The results are presented in the cluster heatmaps. IL-6 and CXCL1 mRNA levels are shown in the histogram on the right. (**E**) The senescence markers p53, p21, and p16 were analyzed by western blotting. The results are presented in the scatter plot. Actin was used as the loading control. (**F**) Immunofluorescence images showing BRD4 (green) and p16 (green). The nuclei were counterstained with DAPI (blue). (**G**) Representative Oil Red O (ORO) staining and statistical data were used to assess lipid uptake. The data all represent measurement data presented as the mean ± SD. The two groups were statistically analyzed using independent sample t-test. One-way ANOVA was used in comparisons among multiple groups, followed by Tukey’s post-hoc test. Significant differences among the different groups are indicated as *p <0.05 vs. LPS; ***p <0.001 vs. LPS; ****p <0.0001 vs. LPS. The experiment was repeated three times.

Next, we sought to determine the contribution of BRD4 to promote senescence of THP-1 macrophages. First, we knocked down BRD4 using a short interfering RNA (siRNA) to reduce the level of BRD4 without altering the levels of BRD2 or BRD3 (Figure 2A, 2B). The low expression of BRD4 attenuated LPS-induced senescence (Figure 2C). Then, we performed quantitative polymerase chain reaction (q-PCR) assays for several genes related to SASP. For instance, we found that the levels of the IL-6 and CXCL1 transcripts increased significantly after treatment with LPS. The increase was reversed by knockdown of BRD4 (Figure 2D). Compared with LPS-induced senescent cells, the knockdown of BRD4 decreased the p53, p21, p16 protein levels (Figure 2E). Similar results were obtained using immunofluorescence (Figure 2F). Furthermore, THP-1 macrophages stained with Oil Red O showed extensive lipid accumulation after LPS stimulation, which was mainly reduced in the presence of BRD4 knockdown. (Figure 2G).

### BRD4 is a novel target for the prevention of macrophage senescence

**Figure 3:**
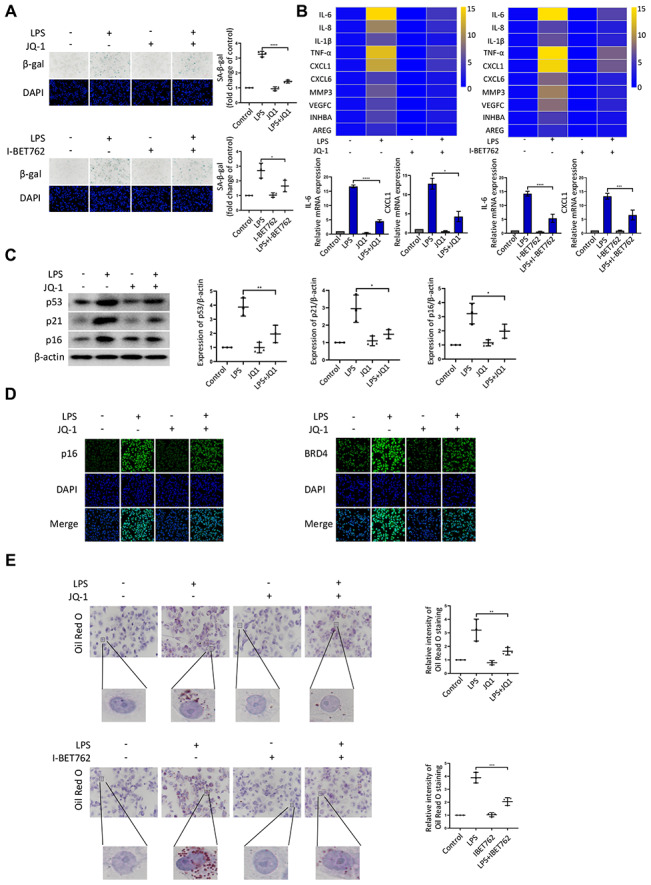
**BRD4 is a novel target for the prevention of macrophage senescence.** THP-1 macrophages were incubated with or without LPS. The cells were then treated with the inhibitors JQ-1 or I-BET762. (**A**) SA-β-gal staining was performed and quantified. (**B**) The mRNA levels of the relative expression of SASP genes are shown in the cluster heatmaps. The histogram on the right shows the exact mRNA levels of IL-6 and CXCL1. (**C**) The protein levels of the senescence markers p53, p21, and p16 were evaluated by western blotting. (**D**) The immunofluorescence of THP-1 cells stained for p16 (green), BRD4 (green), and DAPI (blue) was observed by confocal microscopy. (**E**) Representative ORO staining and statistical data were used to analyze the lipid accumulation of THP-1 macrophages. The data all represent measurement data presented as the mean ± SD. The different groups were statistically analyzed using one-way ANOVA. Significant differences among the different groups are indicated as *p <0.05 vs. LPS; **p <0.01 vs. LPS; ***p <0.001 vs. LPS; ****p <0.0001 vs. LPS. The experiment was repeated three times.

Given that BRD4 was found to be involved in senescence induced by LPS, we used several inhibitors to further characterize the role of BRD4 in the process of aging. JQ-1 and I-BET762 (GSK525762) are both potent BET bromodomain inhibitors. As shown in Figure 3A, LPS stimulation elevated the number of cells positive for β-gal, and JQ-1 and I-BET762 rescued this increase. The mRNA levels of SASP showed a decrease in the cells treated with JQ-1 or I-BET762 after LPS-induced senescence (Figure 3B). Furthermore, we observed a corresponding downregulation in the protein expression of senescence markers p53, p21, and p16 (Figure 3C). Moreover, Immunofluorescence analysis showed the enhanced nuclear staining of p16 in LPS-treated cells in comparison to untreated cells, an effect that was significantly alleviated by JQ-1 treatment (Figure 3D). The Oil Red O staining results showed that lipid accumulation was upregulated in senescent cells, a trend that was attenuated by treatment with JQ-1 or I-BET762 (Figure 3E).

### BRD4 is indispensable for LPS-induced peritoneal macrophage senescence in mice

**Figure 4:**
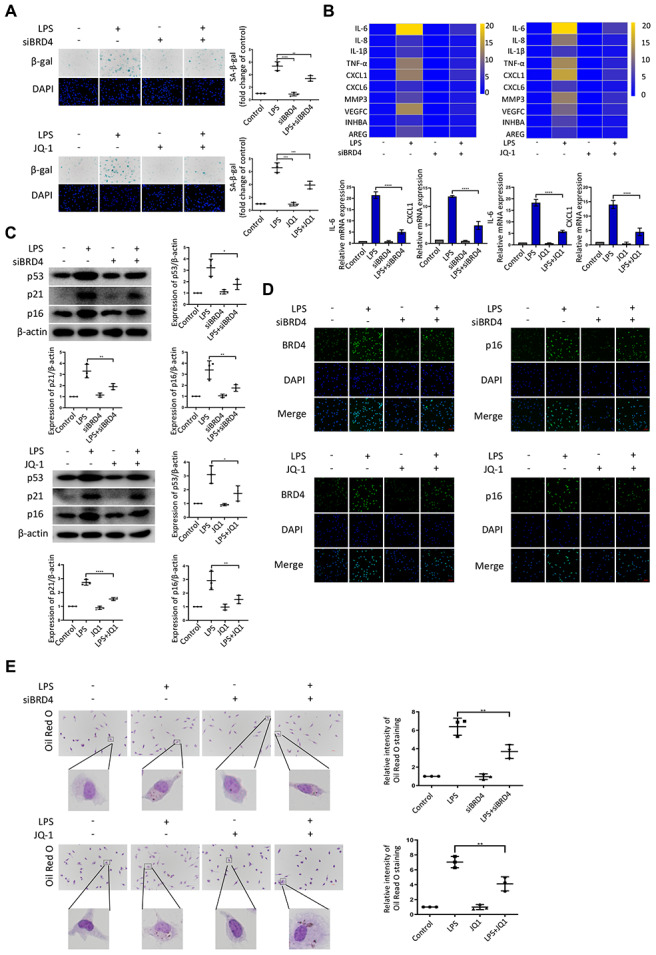
**BRD4 is indispensable for LPS-induced peritoneal macrophage senescence in mice.** Peritoneal macrophages from wild type mice were treated with BRD4-specific siRNA or the BRD4 inhibitor JQ-1 for 24 h, followed by LPS stimulation for 24 h. (**A**) Representative SA-β-gal and DAPI counterstaining of peritoneal macrophages from C57BL6 mice and quantitative analysis of positive cells. Scale bar, 50 μm. (**B**) Cluster heatmaps of SASP transcription levels and scatter plots of representative differentially expressed IL-6 and CXCL1 by qRT-PCR analysis. (**C**) Western blot analysis and quantification of the expression of the senescence-related markers p53, p21, and p16. (**D**) Immunofluorescence measurements of BRD4 and p16 examined by confocal microscopy. Scale bar, 50 μm. (**E**) The lipid accumulation was measured by representative Oil Red O staining. The number of positive results was counted. The data all represent measurement data presented as the mean ± SD. Statistical analysis was performed for the comparison of multiple groups using one-way ANOVA, followed by Tukey’s post-hoc test. The experiment was repeated three times. *p <0.05 vs. LPS; **p <0.01 vs. LPS; ***p <0.001 vs. LPS; ****p <0.0001 vs. LPS.

To further explore the role of BRD4 in the regulation of LPS-induced macrophage senescence, peritoneal macrophages were isolated from wild-type mice. After treatment with LPS, the hallmarks of senescence including senescent phenotype (SA-β-Gal staining), expressions of SASPs, and DNA damage markers were all upregulated (Figure 4A–4C), concurrently with the elevation of BRD4 expression (Figure 4D) in the isolated cells. While BRD4 knockdown or inhibition subsequently reduced the upregulation, it did not reverse it (Figure 4A–4D). Furthermore, the peritoneal macrophage cells stained with Oil Red O showed extensive lipid accumulation after LPS stimulation, where the knocking down of BRD4 resulted in a significant reduction (Figure 4E).

### BRD4 is essential for maintaining the chromatin environment for LPS-induced macrophage senescence

**Figure 5:**
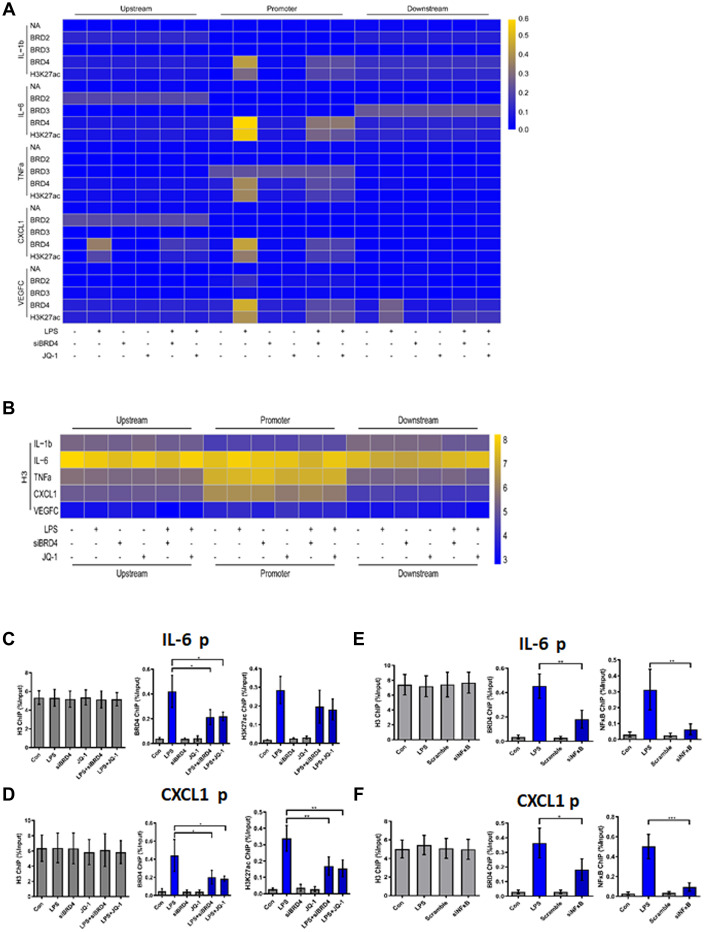
**BRD4 is essential for maintaining the chromatin environment for LPS-induced macrophage senescence.** (**A**) To demonstrate the binding of the indicated proteins to the target genes in THP-1 macrophages, a heatmap was generated using the ChIP-qPCR values. ChIP-qPCR enrichments of no-antibody control (NA) BRD2, BRD3, BRD4, H3K27ac, and H3 using primers spanning –1 kb to +1 kb of the indicated SASP genes were arrayed from blue (no enrichment) to yellow (maximal enrichment). (**B**) The control H3 protein co-precipitated evenly with the ChIP targets. A heatmap was generated using the ChIP-qPCR values for histone H3 between –1 kb and +1 kb of the indicated genes. No significant changes were observed in H3 enrichment for the listed genes in each treatment group. P-values were determined using one-way ANOVA. (**C**, **D**) The representative ChIP-qPCR values for the IL-6 and CXCL1 genes selected from the experiments. (**E**, **F**) Representative ChIP-qPCR values for the IL-6 and CXCL1 genes selected from experiments with different treatments. The horizontal dotted line indicates the upper limit of the 95% confidence interval of the signal from no-antibody (NA) ChIP. Values are presented as the mean ± SEM of three independent experiments. *p <0.05 from one-way ANOVA.

To demonstrate the binding of the indicated proteins (annotated in Figure 5) to their target genes, we performed chromatin immunoprecipitation (ChIP) analysis of the promoter elements (−1 kb to +1 kb) of inflammatory genes in THP-1 macrophages with or without LPS and JQ-1.

ChIP-qPCR was used to analyze no-antibody control (NA), BRD2, BRD3, BRD4, H3K27ac, and H3 (Positive control). No significant changes in the expression of NA, BRD2, or BRD3 for the genes studied were found in the treatment groups. Strikingly, after LPS treatment, the promoter regions of the inflammatory genes in THP-1 macrophages were found to be enriched for BRD4 and H3K27ac (Figure 5A). The cells were then treated with siBRD4 and bromodomain inhibitor JQ-1, respectively, which resulted in the reversal of increased expression of these epigenetic markers. The control marker H3 was pulled down evenly (Figure 5B).

To highlight this novel mechanism of BRD4 oriented chromatin environment for controlling SASP expression, loci of IL-6 and CXCL1 were illustrated to show the BRD4 is essential for SASP expression (Figure 5C, 5D). BRD4 and H3K27ac were analogously enriched at the IL-6 and CXCL1 promoters in cells during LPS-induced senescence, while inhibition of BRD4 either by siBRD4 or JQ1 could prevent BRD4 and H3K27ac from binding on these loci respectively. Interestingly, siNF-κB blocked the binding of BRD4 and H3K27ac on these loci induced by LPS stimulation (Figure 5E, 5F). Together, these results indicated that treatment with LPS increased the expression of SASP by altering the BRD4-mediated histone PTMs dependent on the activation of NF-κB.

### BRD4 promotes the senescence of peritoneal macrophages in senescent mice and human peripheral blood mononuclear cells

**Figure 6:**
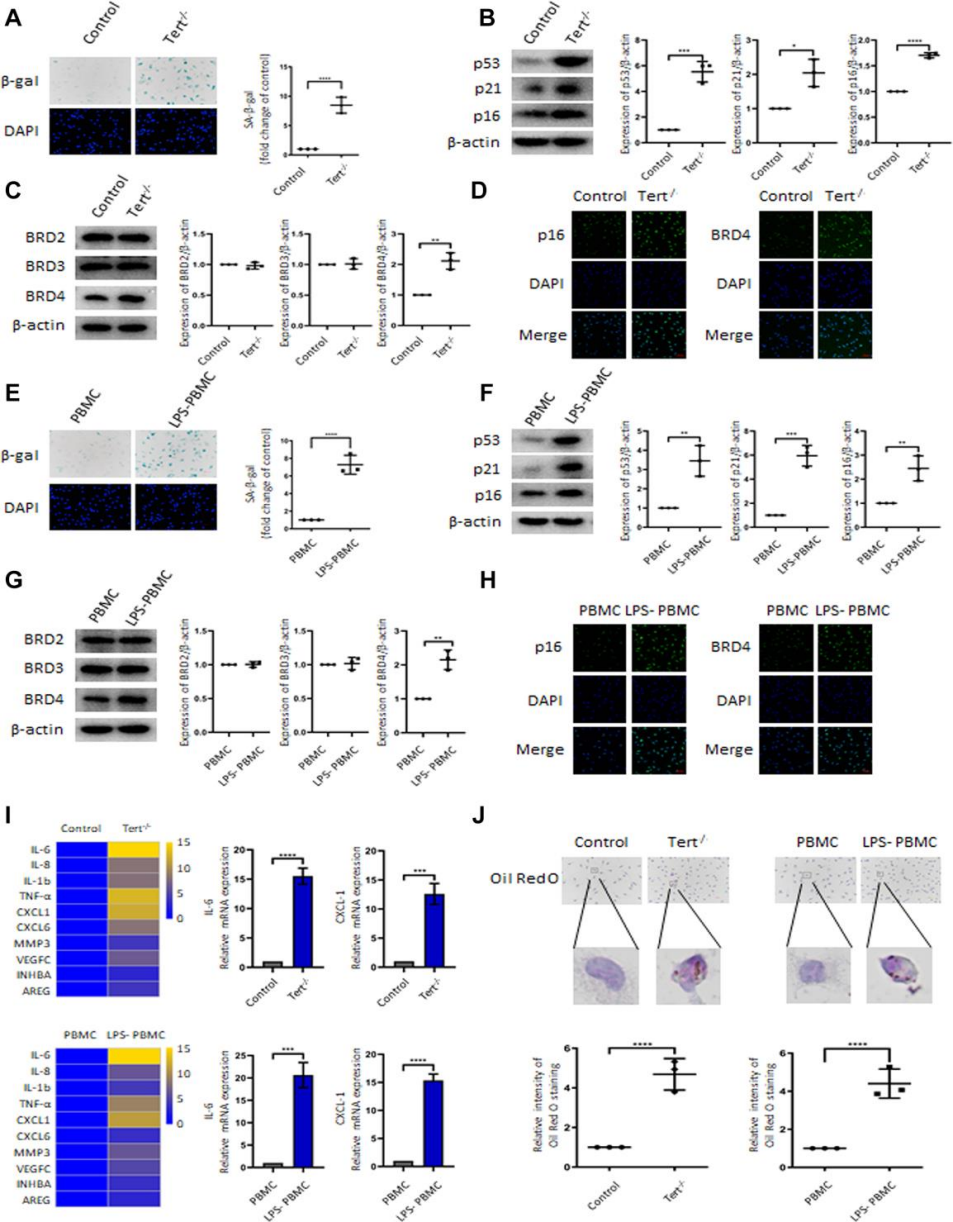
**BRD4 promotes the senescence of mouse peritoneal macrophages and human peripheral blood mononuclear cells.** The primary peritoneal macrophages isolated from Tert^-/-^ senescent mice and human peripheral blood mononuclear cells (PBMCs) were prepared. (**A**, **E**) SA-β-gal staining images and the corresponding quantification in peritoneal macrophages of Tert^-/-^ mice and PBMCs. Scale bar, 50 μm. (**B**, **F**) Western blotting and statistical analysis of protein expression of aging-related markers p53, p21, and p16. (**C**, **G**) Western blot analysis of BRD2, BRD3, and BRD4 in two different cells. (**D**, **H**) Confocal microscopy was used to verify the expression of p16 and BRD4 in peritoneal macrophages of Tert^-/-^ mice and PBMCs. Scale bar, 50 μm. (**I**) qRT-PCR analysis of mRNA expression of SASP in peritoneal macrophages of Tert^-/-^ mice and PBMCs and representative differentially expressed IL-6 and CXCL1. (**J**) Oil Red O staining measured lipid accumulation in peritoneal macrophages and PBMCs. The number of positive results was counted. Scale bar, 50 μm. The data all represent measurement data presented as the mean ± SD. The two groups were statistically analyzed using independent sample t-test. The experiment was repeated three times. Significant differences among the different groups are indicated as *p <0.05 vs. control; **p <0.01 vs. control; ***p <0.001 vs. control; ****p <0.0001 vs. control; **p <0.01 vs. PBMC; ***p <0.001 vs. PBMC; ****p <0.0001 vs. PBMC.

To confirm the involvement of BRD4 in the senescence of mouse peritoneal macrophages in vivo, experiments were performed on peritoneal macrophages isolated from Tert^-/-^ mice and human peripheral blood mononuclear cells. SA-β-gal staining, DNA damage markers’ gene expression (Figure 6A, 6B) confirmed the establishment of senescent-macrophage mouse models. The expression of BRD4 (Figure 6C) and the immunofluorescence staining of p16 and BRD4 (Figure 6D) were increased in the peritoneal macrophages from Tert^-/-^ mice. Consistent with observations from the aging mice model, senescent phenotype and expressions of DNA damage markers were robustly induced in LPS-stimulated human peripheral blood mononuclear cells (PBMCs) (Figure 6E, 6F). Similarly, expressions of BRD4 and p16 were dramatically increased in PBMCs responded to LPS stimulation (Figure 6G, 6H). Furthermore, expression of SASPs and lipid accumulation were elevated both in macrophages in Tert^-/-^ mice and the ones in LPS-stimulated PBMCs (Figure 6I, 6J).

### BRD4-induced inflammation reinforces the senescent phenotype via paracrine pathways

**Figure 7:**
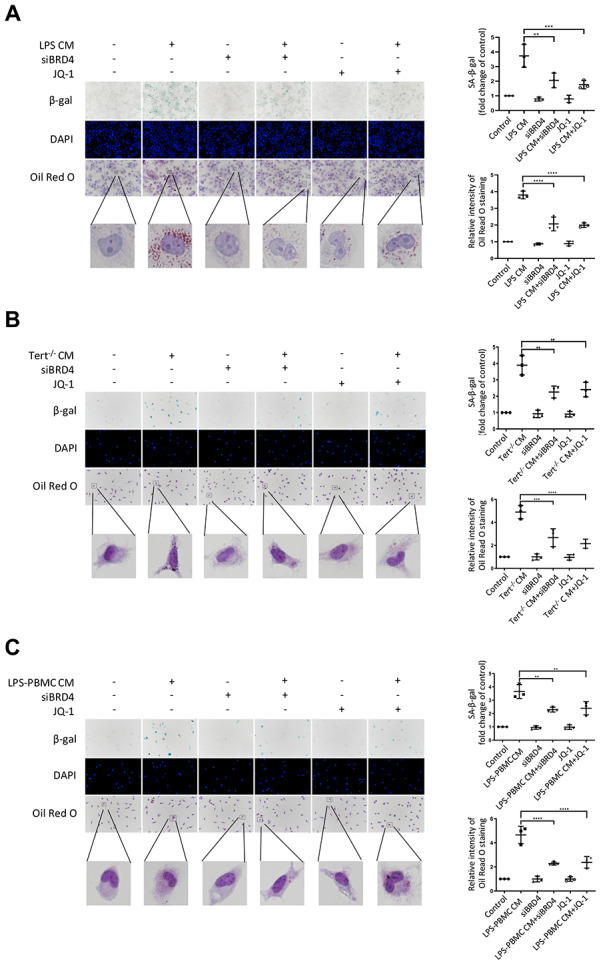
**BRD4-induced inflammation reinforces the senescent phenotype via paracrine pathways.** (**A**) THP-1 macrophages were cultured with LPS-induced senescent cell-derived conditioned medium for 24 h with or without siBRD4 or the BRD4 inhibitor JQ-1 (1 μM). Representative SA-β-gal was used to detect cell senescence, and Oil Red O staining was used to detect lipid accumulation in the cells. Scale bar, 50 μm. (**B**, **C**) The peritoneal macrophages from the Tert^-/-^ mice and human peripheral blood mononuclear cells (PBMCs) were cultured with the corresponding conditioned medium for 24 h. Representative SA-β-gal was used to detect cell senescence, and Oil Red O staining was used to detect lipid accumulation in the cells. Scale bar, 50 μm. The data all represent measured data presented as the mean ± SD. Comparisons between multiple groups were performed using one-way ANOVA, followed by Tukey’s post-hoc test. The experiment was repeated three times. Significant differences among different groups are indicated as **p <0.01 vs. LPS CM; ***p <0.001 vs. Tert^-/-^ CM; ****p <0.0001 vs. LPS-PBMC CM.

Numerous evidence indicated that paracrine signals from senescent cells might foster macrophage senescence and the progression of aging-related diseases. In this study, we explored whether BRD4 is also involved in the circuit of paracrine enlarged senescence circumstances. As we observed, the SA-β-gal activity was found to be visibly enhanced after LPS CM (conditional medium from 24h LPS-stimulated THP-1 macrophages) treatment in THP-1 cell line, suggesting that the paracrine mechanism may underlie macrophage senescence (Figure 7A). We observed that BRD4 knockdown or inhibition reduced the level of SA-β-gal activity, although they were unable to abolish the development of senescence (Figure 7A). In addition, Oil Red O staining demonstrated an increase in intracellular lipid uptake in the LPS-CM group, whereas a decline was observed in LPS CM+siBRD4 and LPS CM+JQ-1 groups (Figure 7A). Similarly, we obtained Tert^-/-^ CM (conditional medium from cultured macrophages from Tert^-/-^ mice) and LPS-PBMC CM (conditional medium from LPS-induced cultured PBMC) by incubating peritoneal macrophages from Tert^-/-^ mice and human peripheral blood respectively. The peritoneal macrophages obtained from wild mice were incubated with Tert^-/-^ CM and normal PBMC were incubated with LPS-PBMC CM respectively. We observed both Tert^-/-^ CM and LPS-PBMC CM could induce senescence in the individually treated cells (Figure 7B, 7C), while interference of BRD4 by knocking down BRD4 or enzymatic-inactivation with JQ-1 can prevent the propagating process of senescence (Figure 7B, 7C). The results of SA-β-gal and Oil Red O analyses further strengthened the verification of BRD4’s function of preventing the Tert^-/-^ CM or LPS-PBMC CM to induce the senescence in macrophages via paracrine pattern (Figure 7B, 7C).

## DISCUSSION

**Figure 8:**
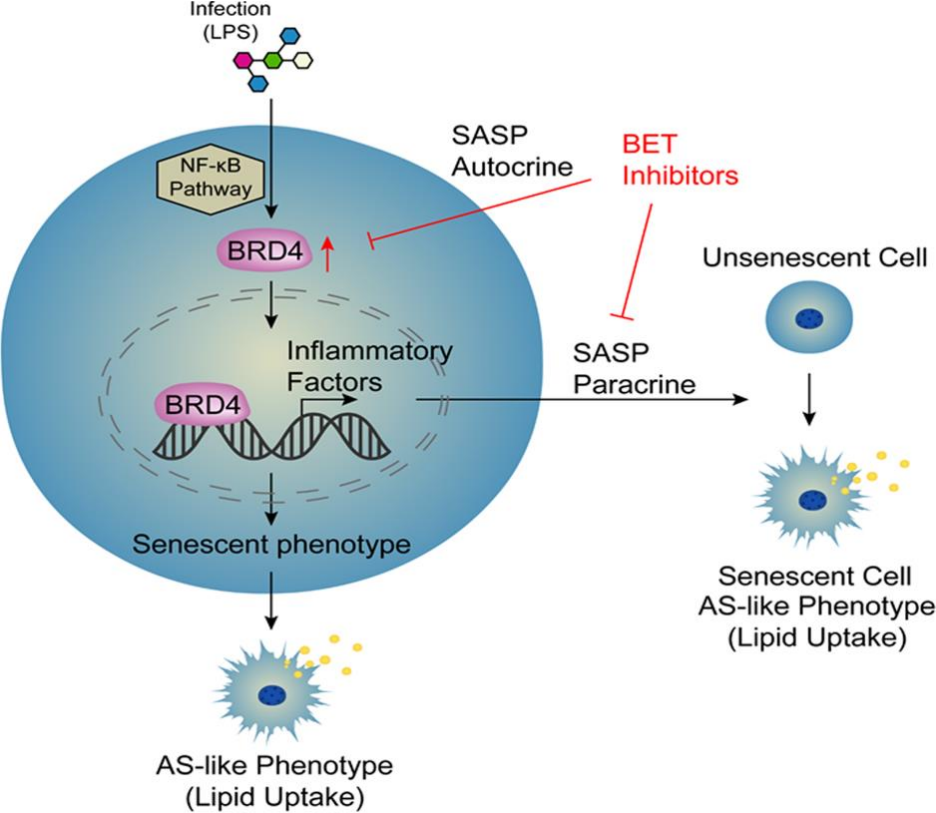
**Model.**

Atherosclerosis is an aging-related disease. Numerous evidence has indicated that cell aging is responsible for promoting the progression of atherosclerosis. Therefore, cellular aging is an important risk factor for atherosclerosis [9]. However, the specific mechanisms of this pathological process have not yet been elucidated. Macrophage activation is a key factor in the development of atherosclerosis [28]. In this study, we collected a variety of macrophages of different origins, including THP-1 macrophages, C57BL6 mouse peritoneal macrophages, and human peripheral blood mononuclear cells (PBMCs), which were stimulated with LPS to establish several novel models of infection-induced senescent macrophages. These models were used to observe the induction of cellular senescence by treatment with LPS through increased expression of BRD4, promoting the production of SASP and atherosclerosis-like progression. After infection, the cells had increased NF-κB-dependent BRD4 expression, which promoted the expression of inflammatory factors and ultimately induced macrophage senescence via the autocrine pathway. At the same time, we used conditional medium from LPS-induced cells to stimulate cells and found that cells became senescent via the paracrine pathway. In addition, siBRD4 and BRD4 inhibitors, such as JQ-1 and I-BET762, could protect the cells against LPS-induced senescence, indicating that BRD4 is a potential drug target for atherosclerosis and aging-related diseases (Figure 8). Importantly, BRD4 is increased in macrophages from Tert^-/-^ mice compared to the wild ones. Meanwhile, the inhibition of BRD4 by siBRD4 or inhibitors can prevent the propagation of senescence from elder cells to younger cells via blocking the paracrine SASP expression. Novel mechanisms of BRD4 involved in the autocrine or paracrine senescence *in vivo* will be further investigated.

Macrophages play a crucial role in age-related diseases via affecting inflammation, phagocytosis, maintaining tissue homeostasis, tissue remodeling, and repair [9]. Macrophage dysfunction leads to abnormal neovascular proliferation in aging-related diseases including cancers, atherosclerosis and blinding eye disease [28]. Recent studies have demonstrated a significant proportion of p16Ink4a/SAβG-positive cells accumulating in aging mice are macrophages [29], which have the same right as senescent cells to be considered a possible contributor to aging [30]. Meanwhile, the bacterial is associated with macrophage activation, aging-related diseases, and atherosclerosis. LPS is able to activate the mononuclear macrophage system by stimulating a wide range of cytokines, such as tumor necrosis factor (TNF), interleukin-6 (IL-6), interferons, and colony-stimulating factors [31]. Several infectious models of LPS were used in this study. Here, we successfully established an LPS-induced senescent model with the hallmarks of senescence, including morphological changes, persistent DNA damage response (DDR), and an aging-related secretory phenotype (SASP) [32].

Previous studies have demonstrated that paracrine signals from senescent cells play a contributory role in aging-related diseases [33]. SASP mediates the paracrine activity of senescent cells through the secretion of multiple factors, including cytokines, chemokines, and growth factors. Intriguingly, senescence can be transmitted to bystander cells by the SASP, resulting in an increased number of senescent cells and perpetuation of the chronic, low-grade, age-related inflammatory state that researchers have previously defined as “inflammaging” [34]. In this study, using conditioned medium derived from LPS-induced senescent cells to stimulate normal cells, the treated cells were found to undergo aging, indicating that aging models exist in both the paracrine and autocrine systems. Therefore, the expression of macrophage pro-inflammatory factors can be controlled through the autocrine/paracrine loop, which ultimately delays aging [35]. Our results demonstrate that aging and lipid accumulation in the LPS-induced senescent models could be ameliorated with blunting SASP expression of autocrine and paracrine. This potential regulatory circuit for the secretion of inflammatory factors could allow for the development of drug targets for aging-related diseases.

BET proteins have a crucial role in regulating gene transcription by recruiting proteins to form complexes that modify chromatin [36]. The transition of RNA Polymerase II (Pol II) from promoter-proximal pausing to productive elongation has emerged as a key rate-limiting step in the expression of almost all active genes [36, 37]. Previous studies have suggested that BRD4 plays a contributory role in releasing promoter-proximally paused RNA Pol II for productive transcription elongation [38].

BRD4 is a conserved member of the bromodomain and extra-terminal (BET) family of chromatin readers, which is closely related to inflammation activation in multiple diseases, such as rheumatoid arthritis, pulmonary arterial hypertension, and chronic obstructive pulmonary disease. Especially, a key role of BRD4 in cardiovascular diseases has recently been extensively investigated. Previous studies indicated that BRD4 acted as a central co-activator of transactivation of pathological genes during cardiac hypertrophy [26]. Furthermore, BRD4 could serve as a new target for regulating the phenotype of profibrotic cardiac fibroblasts and provide a potential therapeutic window suitable for the treatment of chronic fibrotic diseases such as heart failure [38]. In this study, we demonstrated that BRD4 mediates the pathological process of senescent macrophage, which is implicated in aging-related atherogenesis as an emerging therapeutic target.

Finding direct targets of transcription factors and regulatory pathways is a key factor in understanding their role in pathological processes. In fact, BRD4 is a key factor controlling the expression of inflammatory genes. Substantial evidence has addressed various approaches in which BRD4 interacts with transcriptional regulators to nuance transcription [26]. NF-κB activation is a response to a variety of extracellular stimuli, and inflammation-related NF-κB signaling pathways are essential in the development of atherosclerosis [38]. It may be related to the direct interaction between BRD4 and acetylated histones [39]. Evidence suggests that BRD4 can also co-activate pro-inflammatory genes that depend on NF-κB transcription by interacting with acetylated RELA [40]. The formation of NF-κB-directed super-enhancers leads to the overall reorganization of the BRD4 super-enhancers landscape and induces the transcription of many classic pro-inflammatory endothelial genes [41]. Meanwhile, it is also found YAP/TAZ physically participates in the common co-activator BRD4, which mediates the recruitment of BRD4 and RNA Polymerase II (Pol II) at YAP/TAZ-regulated promoters, thereby enhancing the expression of many growth-regulating genes [42]. Therefore, BRD4 can be a target for precise control of these abnormal pathways activation.

BET protein inhibition is an epigenetic regulation strategy that has been used to target inflammatory, cancer and metabolic diseases [35]. BET inhibitors are promising anticancer drugs, which can suppress elusive cancer targets [43]. JQ-1 is a novel small molecule inhibitor that competitively inhibits the binding of the amino-terminal bromine domain in chromatin to acetylated histones, showing anti-inflammatory and anti-cancer activity [44]. It has been reported that I-BET can inhibit the inflammatory response of activated macrophages by interfering with the binding of BET protein to acetylated histones, which is essential for initiating mRNA transcription of inflammatory genes [45]. Meanwhile, the inhibition of BRD4 with small molecules can reduce heart failure progression by regulating specific transcriptional programs [43]. Our investigation has demonstrated that BET inhibitors, by JQ1, I-BET762 or siBRD4 can reduce SASP and delay atherosclerosis progression. In addition, the non-transcriptional role of BRD4 in controlling DNA damage checkpoint activation and repair and telomere maintenance has been proposed, thus providing new ideas for the multiple functions of the protein [46].

Altogether, we demonstrate for the first time that the formation of senescent macrophage in the BRD4-dependent epigenetic manner is one of the key contributors driving the initiation and progression of atherosclerosis. The redistribution of BRD4 on chromosome promoting senescent related protein and SASP gene expression which is regulated by activation of NF-κB. Therefore, developing novel inhibitors targeting BRD4 could be a promising therapeutic strategy of atherosclerosis in clinical via preventing macrophage senescence.

## MATERIALS AND METHODS

### Isolation and culture of murine primary peritoneal macrophages and human mononuclear cells

All C57BL6 mice were purchased from The Jackson Laboratory. Tert^-/-^ mice were provided by Professor Qigang Zhou's laboratory at Nanjing Medical University. All animal experiments were approved by the Animal Care and Use Committee of Nanjing Medical University and were performed in accordance with the corresponding institutional guidelines. Briefly, the mice were euthanized by cervical dislocation and the abdominal skin was removed to expose the peritoneum. Subsequently, 15 ml PBS buffer (Origene, China) was injected into an abdominal cavity. Peritoneal macrophages in the peritoneal lavage fluid were aseptically collected by centrifugation for 6 min at 1200 rpm. The cell pellet was collected and resuspended in RPMI 1640 medium (HyClone, USA) with 10% fetal bovine serum (FBS) (Gibco, USA) and 1% penicillin and streptomycin (Beyotime, China). After incubating for 2 h in a humidified 5% CO2 atmosphere at 37°C, the nonadherent cells were removed, and the peritoneal macrophages were obtained. For the human samples, we obtained 30 ml of peripheral blood from researchers in the study with informed consent and separated peripheral blood mononuclear cells (PBMCs). The cells were then incubated as described above.

### Cell culture and treatment

THP-1 human monocyte cells, murine primary peritoneal macrophages, and human peripheral blood mononuclear cells were cultured in 25 mM HEPES-buffered RPMI 1640 supplemented with 10% heat-inactivated FBS, 1% penicillin, and streptomycin at 37°C with 5% CO2 level in a humidified incubator. After incubating with 100 ng/mL 4β-phorbol-12-myristate-13-acetate (PMA) (La Jolla, USA) for 24 h at a density of 1.0×10^5^ cells/mL, the THP-1 cells differentiated into macrophages. For cell treatment, THP-1 macrophages, peritoneal macrophages, and PBMCs were stimulated with Lipopolysaccharide (LPS) (1 μg·ml^−1^) for 24 h to induce senescence. Cells were treated with BET inhibitors, JQ-1 (1 μM) (Selleck, USA) and I-BET762 (0.5 μM) (Selleck, USA), to prevent LPS-induced senescence.

### Small interfering RNA

Cells were transfected with small interfering RNA (siRNA) or negative control siRNA (Shanghai GenePharma, China) using Lipofectamine 3000 Reagent (Invitrogen, USA) according to the manufacturer’s instructions. Briefly, the Lipofectamine 3000 Reagent and siRNAs were diluted with serum-free RPMI 1640 and incubated for 5 min. The diluted reagents were then mixed, incubated for 15 min at room temperature, and added to the cells. After 4-6 h, the medium was changed with RPMI 1640 containing 10% FBS and the cells were incubated with fresh medium for 20-18 h before stimulation. The sequences of the siRNAs used were as follows: BRD4-Homo-2324 (5’-GGAAACCUCAAGC UGAGAATT-3’) and NF-κB (5’-TATTAGAGCAACC TAAACATT-3’).

### Western blotting

Whole-cell proteins were extracted using RIPA (Beyotime, China) buffer containing the protease inhibitor phenylmethylsulfonyl fluoride (PMSF) (Beyotime, China). A BCA Protein Assay Kit was used to quantify the protein. The proteins were then mixed with 6× Loading Buffer before denaturing by boiling for 5 min at 100°C. The mixtures were then separated using sodium dodecyl sulfate–polyacrylamide gel electrophoresis (SDS-PAGE) and transferred onto polyvinylidene difluoride membranes. The membranes were blocked and incubated with the specific primary antibodies at 4°C overnight. After washed with TBS-T (Mdbio, China), the membranes were incubated with the appropriate secondary antibody for 1 h at room temperature. The immunoreactive bands were visualized using Pierce ECL Western Blotting Substrate and analyzed using Image J software. The specific protein expression levels of the blots were normalized to β-actin (Abcam, USA). The antibodies used for blotting are listed in Supplementary Table 1.

### RNA extraction and qRT-PCR

Total RNA was extracted from the cells using Trizol reagent (Invitrogen, USA) according to the manufacturer’s instructions. After reverse transcription with HiScript-II-Q RT SuperMix for qPCR (Vazyme, Nanjing, China), real-time PCR was performed using an ABI 7500 Real-Time PCR System (Applied Biosystems, Foster City, CA, USA). The raw data (Ct values) were analyzed using the comparative Ct method. All of the primers were synthesized by a commercial vendor (Nanjing Generay, China). The PCR primer sequences are provided in Supplementary Table 2.

### Immunofluorescence

After different treatment, the cells seeded in glass-bottomed culture dishes were fixed in 4% paraformaldehyde for 20 min, followed by permeabilized with 0.5% Triton X-100 for 20 min. After blocked with 3% BSA for 1 h, the cells were incubated with the specific primary antibody against BRD2, BRD3, BRD4, and NF-κB (p65) overnight at 4°C. Then, the cells were incubated with the secondary antibody for 1 h at room temperature and counterstained with DAPI to track nucleus. The images of the labeled cells were performed by the confocal laser scanning microscope (LSM800; Zeiss, Oberkochen, Germany).

### Senescence-associated β-galactosidase (SA-β-Gal staining) activity

SA-β-Gal staining was performed as previously described with slight modification [32]. The cells were fixed and incubated overnight at 37°C using a Senescence β-Galactosidase Staining Kit (Beyotime Biotechnology, China) according to the manufacturer's instructions. Following the mounting of the coverslips, fluorescence images were detected under a fluorescence microscope (Nikon, Japan) at a magnification of 20× for the SA-β-gal-positive cells.

### Oil Red O staining

After stimulated with LPS for 24 h, the culture medium was changed for fresh medium containing 50 μg·ml^−1^ of ox-LDL (Peking Union Bio, China). To analyze the lipid accumulation, the cells after different treatments were fixed by 4% paraformaldehyde and dehydrated in 60% isopropanol for 2 min. Then, the cells were stained with filtered 0.3% Oil Red O (Abcam, USA) for 30 min and hematoxylin (Jiancheng Biotech, China) for 1 min. Finally, wash of PBS buffer, the cells washed by PBS buffer and were examined by light microscopy to observe the lipid accumulation in macrophages.

### Chromatin immunoprecipitation (ChIP)

ChIP was performed as previously described with slight modification [47]. Cells were cross-linked with 1% formaldehyde in PBS for 10 min and quenched by the addition of 2.5 M glycine for 5 min at room temperature. Chromatin fragments ranging from 300 to 700 bp were collected by ultrasonication in lysis buffer (50 mM Tris, pH 7.9, 10 mM EDTA, 1% SDS, protease-inhibitor cocktail, and 1 mM DTT). The supernatant was obtained by centrifugation at 14,000 rpm and 4°C for 10 min. Then, 20 μl was used as the input for quantification. The remaining supernatant was diluted ten-fold in dilution buffer (20 mM Tris, pH 7.9, 2 mM EDTA, 150 mM NaCl, 0.5% Triton X-100, protease-inhibitor cocktail, and 1 mM DTT) and pre-cleared with 20 μl 50% protein A-agarose beads at 4°C for 1-3 h. The supernatant was immunoprecipitated at 4°C overnight with no-antibody or antibodies against BRD2, BRD3, BRD4, H3K27ac, NF-κB and H3. The following day, the mixture was incubated with 40 μl 50% protein A-agarose beads at 4°C for 2 h. The beads were then washed with wash buffer (20 mM Tris, pH 7.9, 2 mM EDTA, 0.05% SDS, 250 mM NaCl, and 0.25% Triton X-100), resuspended with elution buffer, and boiled at 65°C overnight. No-antibody (NA) controls were performed as in the other groups. On the third day, Txn stop solution containing 1mg·ml^−1^ glycogen and 0.05 mg·ml^−1^ proteinase K was used to digest the protein of immunoprecipitated chromatin at 37°C for 1 h. The DNA was then extracted with phenol: chloroform: isoamyl alcohol (25:24:1), ammonia acetate, and ethanol. The enrichment of the immunoprecipitated material was analyzed by qRT-PCR using the percentage input method. The antibodies used for ChIP are listed in Supplementary Table 3.

### Statistical analysis

All data were presented as the median or mean ± standard error of the mean (SEM) or standard deviation (SD). The Student’s t-test was used for comparisons between experimental groups. ANOVA was used for multiple comparisons, followed by the Sidak post-test. Data were analyzed using the Prism software v6 (GraphPad Software Inc.); *p*-values <0.05 were considered significant (*p <0.05; **p <0.01; ***p <0.001; ****p <0.0001).

## Supplementary Material

Supplementary Tables
