# Correction for: BRD4 contributes to LPS-induced macrophage senescence and promotes progression of atherosclerosis-associated lipid uptake

**DOI:** 10.18632/aging.206283

**Published:** 2025-07-31

**Authors:** Hui Wang, Haiping Fu, Ruigong Zhu, Xuan Wu, Xian Ji, Xuesong Li, Hong Jiang, Zhe Lin, Xin Tang, Shixiu Sun, Jiajing Chen, Xin Wang, Qingguo Li, Yong Ji, Hongshan Chen

**Affiliations:** 1 Key Laboratory of Cardiovascular and Cerebrovascular Medicine, School of Pharmacy, Nanjing Medical University, Nanjing, China; 2 Department of Cardiothoracic Surgery, The Second Affiliated Hospital of Nanjing Medical University, Nanjing, China; 3 Key Laboratory of Targeted Intervention of Cardiovascular Disease, Collaborative Innovation Center for Cardiovascular Disease Translational Medicine, Nanjing Medical University, Nanjing, China; 4 Faculty of Biology, Medicine and Health, The University of Manchester, Manchester, United Kingdom

**Keywords:** inflammation, senescence, BRD4, macrophage, gene expression

**This article has been corrected:** The authors have identified two inadvertent errors in **Figure 7B**. Specifically, the same representative Oil Red O image was used for both Figure 7C (labeled “LPS-PBMC CM+JQ-1”) and Figure 7B (labeled “JQ-1”). Additionally, in Figure 7B, the β-gal image labeled “siBRD4+Tert−/− CM” partially overlapped with the β-gal image labeled “siBRD4+LPS-PBMC CM” in Figure 7C. The author provided the original images for Figure 7B from the original experiments and confirmed that these errors do not compromise other results. The incorrect images have been replaced with the correct ones from the original experiments. This correction has no impact on the experimental outcome or conclusions. The authors sincerely apologize for this error.

The corrected version of **Figure 7** is provided below.

**Figure 7:**
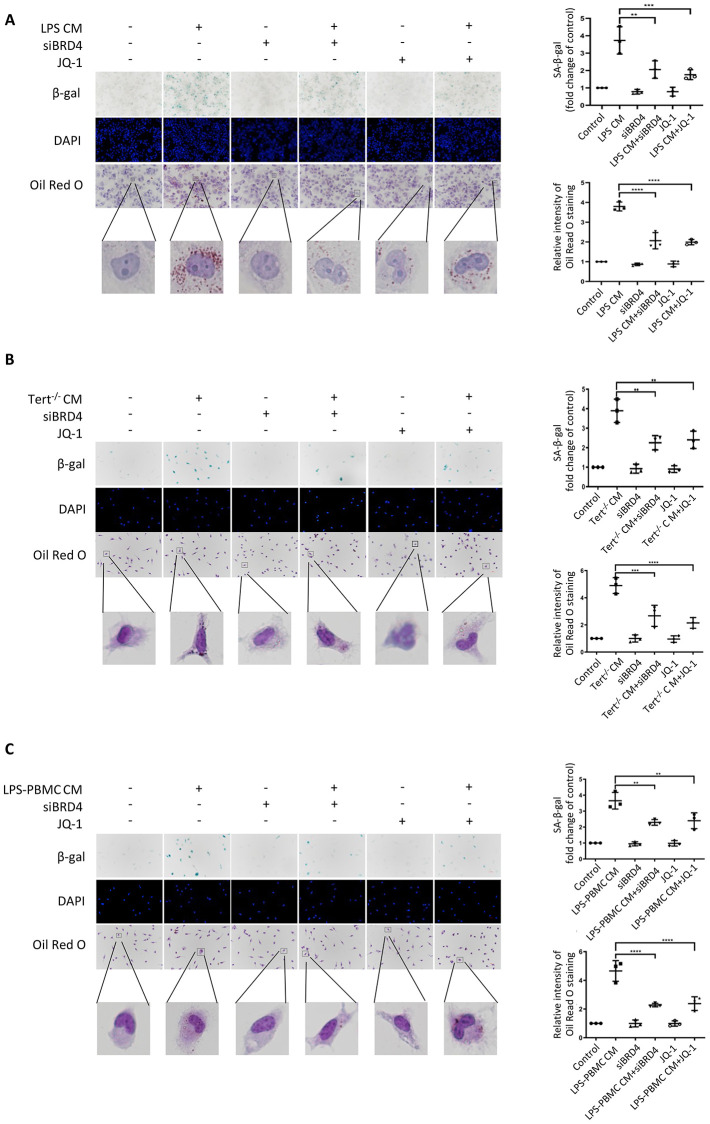
**BRD4-induced inflammation reinforces the senescent phenotype via paracrine pathways.** (**A**) THP-1 macrophages were cultured with LPS-induced senescent cell-derived conditioned medium for 24 h with or without siBRD4 or the BRD4 inhibitor JQ-1 (1 µM). Representative SA-β-gal was used to detect cell senescence, and Oil Red O staining was used to detect lipid accumulation in the cells. Scale bar, 50 µm. (**B**, **C**) The peritoneal macrophages from the Tert^−/−^ mice and human peripheral blood mononuclear cells (PBMCs) were cultured with the corresponding conditioned medium for 24 h. Representative SA-β-gal was used to detect cell senescence, and Oil Red O staining was used to detect lipid accumulation in the cells. Scale bar, 50 µm. The data all represent measured data presented as the mean ± SD. Comparisons between multiple groups were performed using one-way ANOVA, followed by Tukey’s post-hoc test. The experiment was repeated three times. Significant differences among different groups are indicated as ^**^p < 0.01 vs. LPS CM; ^***^p < 0.001 vs. Tert^−/−^ CM; ^****^p < 0.0001 vs. LPS-PBMC CM.

