# Supplementary material for: BRD4 contributes to LPS-induced macrophage senescence and promotes progression of atherosclerosis-associated lipid uptake
Source: Aging (Albany NY). 2020 May 11;12(10):9240–59. doi: 10.18632/aging.103200 (PMC7288959; doi:10.18632/aging.103200)
Supplement: Supplementary Tables [file aging-12-103200-s001..pdf]

SUPPLEMENTARY TABLES

Supplementary Table 1. Antibodies used in this study.

Factor or PTM	Vendor	Cat number	Dilution for blotting	Vol.for ChIP (µl)
BRD2 (for human)	Abcam	ab139690	1:1,000	8
BRD2 (for mouse)	Abcam	ab3718	1:1,000	
BRD3	Bethyl	A302-368A	1:1,000	8
BRD4	Bethyl	A700-004	1:1,000	5
H3K27ac	Abcam	ab4729	1:1,000	5
H3	Abcam	ab1791	1:3,000	8
P53	Abcam	ab131442	1:1,000	
P21	Abcam	ab109199	1:1,000	
P16INK4	Abcam	ab189034	1:1,000	
NFκB	GeneTex	GTX102090	1:1,000	5
p-NFκB	GeneTex	GTX55114	1:1,000	
β-Actin	Abcam	ab8227	1:1,000	
HRP-conjugated goat anti-rabbit secondary antibody	Jackson Labs	111035003	1:30,000	
HRP-conjugated goat anti-mouse secondary antibody	Jackson Labs	115036003	1:30,000	

Supplementary Table 2. RT-PCR primers used in this study (human).

Gene name	Forward sequence	Reverse sequence
IL6	TTCTGCGCAGCTTTAAGGAG	AGGTGCCCATGCTACATTTG
IL8	ATGACTTCCAAGCTGGCCGTG	TGTGTTGGCGCAGTGTGGTC
CXCL1	CACCCCAAGAACATCCAAAG	TAACTATGGGGGATGCAGGA
CXCL6	TGTTTACGCGTTACGCTGAG	AACTTGCTTCCCGTTCTTCA
VEGFC	AGAGAACAGGCCAACCTCAA	TGGCATGCATTGAGTCTTTC
INHBA	CGGCGCTTCTGAACGCGATC	GCTGTTCCTGACTCGGCAAACGT
MMP3	AGGGAACTTGAGCGTGAATC	TCACTTGTCTGTTGCACACG
AREG	AGCTGCCTTTATGTCTGCTG	TTTCGTTCCTCAGCTTCTCC
IL1β	TGCACGCTCCGGGACTCACA	CATGGAGAACACCACTTGTTGCTCC
BRD2	GGAAACATCAGTTCGCATGGC	CACTCTGAAGCAGCCCAATAA
BRD3	TGCAAGCGAATGTATGCAGGA	CATCTGGGCCACTTTTTGTAGAA
BRD4	ACAACCCTCCTGACCATGAG	AACTGTCACTGTCCGAGGAG
β-Actin	CTACCTCATGAAGATCCTCACCGA	TTCTCCTTAATGTCACGCACGATT

RT-PCR primers used in this study (mouse).

Gene name	Forward sequence	Reverse sequence
IL6	TTCCATCCAGTTGCCTTCTT	CAGAATTGCCATTGCACAAC
IL8	CACCCTCTGTCACCTGCTCAA	ATGGCGCTGAGAAGACTTGGT
CXCL1	TGCACCCAAACCGAAGTCAT	TTGTCAGAAGCCAGCGTTCAC
CXCL6	CGGTCCTGCTCGTCATTCAC	CGTAGCTCCGTTGCAACCAT
VEGFC	CAAGGTCGGGCAGGAAGAG	TAGAAGGCACAGTCGAGG
INHBA	TGAATGAACTCATGGAGCAGACC	AGCTGGCTGGTCCTCACAG
MMP3	ATGAAAATGAAGGGTCTTCCGG	GCAGAAGCTCCATACCAGCA
AREG	CTTTGGTGAACGGTGTGGAG	TCGTTTCCAAAGGTGCACTG
IL1β	CTCTCCAGCCAAGCTTCCTTGTGC	GCTCTCATCAGGACAGCCCAGGT

BRD2	CCACGAAAAGACTTGCCTGA	CAGCGTGCTTCTTTGAGAGC
BRD3	CTATGCGTGGCCCTTTTACA	CTTCCTTTTGACCGTGCTGA
BRD4	CAAAAGGAAGAGGACGAGGG	ACAGGTGGAGGAGGGTTCTG
β-Actin	GGCTGTATTCCCCTCCATCG	CCAGTTGGTAACAATGCCATGT

Supplementary Table 3. ChIP primers used in this study.

Primer set name	Forward sequence	Reverse sequence
IL1β upstream	AACCGAGACACCAGCAAAGT	GCAGACCTGTCAAAGAGGCA
IL1β promoter	GAATCCCAGAGCAGCCTGTT	AACAGCGAGGGAGAAACTGG
IL1β downstream	GCCTCTCAAAGCTGCCTGAA	CCTGCCAGGCCTAGAATCTG
IL6 upstream	ATTGGGAGACCAGCTCATTG	CAGCTCGGCTATATCGGTTC
IL6 promoter	GATTCCTCAAAGCCATTCCA	TGGAGTCCAGAGGTGGTAGG
IL6 downstream	GAAAGCAGCAAAGAGGCACT	TGCGACAAGTTGGTACCTCA
TNFα upstream	GTGTATGTCAGTGTGTTCCAGTCTT	GCTGGGAGGGATCTGCTCCTCTCTC
TNFα promoter	GGGAAACCCCAGGGAAAGAA	TATAAACGCTGGCAGGGGAT
TNFα downstream	GGGTTAAGGGGAGCAAAGTCAGAT	CTGGGGAAAGGAGGCACAAAGAAG
CXCL1 upstream	TGGAAACTGAGCTTTTGGTG	TGCTACCCAACTACCCCTAATG
CXCL1 promoter	CAACGCTCTTCCTCCAAAGA	CTGCGCTGAAGATACCACCT
CXCL1 downstream	ATTTCTGAGGAGCCTGCAAC	GATCTCATTGGCCATTTGCT
VEGFC upstream	CTTCCTTACCCCCTGGAAAC	AGGTCTTGCATGAAGCTAGTGA
VEGFC promoter	CTCACAGGAAACCGGACATC	CGCCTCTCCAAAAAGCTACA
VEGFC downstream	CACAGGTACCTCTCCACTTGC	ACACTTGCAGTACCCCTTGC
